# Heat or Burn? Impacts of Intrauterine Tobacco Smoke and E-Cigarette Vapor Exposure on the Offspring’s Health Outcome

**DOI:** 10.3390/toxics6030043

**Published:** 2018-08-01

**Authors:** Gerard Li, Sonia Saad, Brian G. Oliver, Hui Chen

**Affiliations:** 1 School of Life Sciences, University of Technology Sydney, Sydney, NSW 2007, Australia; Gerard.E.Li@student.uts.edu.au (G.L.); sonia.saad@sydney.edu.au (S.S.); Brian.Oliver@uts.edu.au (B.G.O.); 2 Renal Group, Kolling Institute, Royal North Shore Hospital, St Leonards, NSW 2065, Australia; 3 Respiratory Cellular and Molecular Biology, Woolcock Institute of Medical Research, Sydney, NSW 2037, Australia

**Keywords:** maternal smoking, e-cigarette, offspring, heat-not-burn tobacco, nicotine-free

## Abstract

Maternal smoking during pregnancy leads to gestational complications and organ disorders in the offspring. As nicotine replacement therapy is often ineffective for smoking cessation, pregnant women turn to alternatives such as heat-not-burn tobacco and e-cigarettes. Recently, the popularly of e-cigarettes has been increasing especially among the youth and pregnant women, mainly due to the advertisements claiming their safety. This has even led to some clinicians recommending their use during pregnancy. E-cigarettes heat e-liquid to produce an aerosol (e-vapor), delivering flavorings and nicotine to the user. However, e-vapor also contains toxins such as formaldehyde along with heavy metals and carcinogenic nitrosamines. In addition, specific flavoring compounds such as diacetyl can be toxic themselves or decompose into toxic compounds such as benzaldehydes. These compounds can induce toxicity, inflammation and oxidative stress in the mothers and can accumulate in the developing fetus, affecting intrauterine development. Recent animal studies suggest that maternal e-vapor exposure during pregnancy could cause respiratory and neurological disorders in the offspring. This review will examine the available literature to shed light on the current understanding of this problem-to-be from lessons learned in animal models.

## 1. Introduction

Tobacco cigarette smoking is the primary preventable cause of morbidity and mortality, causing the premature death of over 7 million individuals every year [1]. The World Health Organization estimates that the adult tobacco smoking rate is 14.8% in Australia, 21.9% in the US, 20.8% in the UK, 29.4% in Europe and 25.2% in China in 2016 [2]. Cigarettes burn tobacco to deliver smoke to the user which contains over 4000 chemicals, 93 of which have been regarded as harmful or potentially harmful by the Food and Drug Administration [3]. The components of cigarette smoke are absorbed through the lungs into the bloodstream and thus have the potential to cause diseases in almost all human organ systems and decreases overall health status [4]. Maternal smoking during pregnancy causes a range of negative pregnancy outcomes, including: miscarriage, low birth weight, preterm birth, and perinatal death [5]. Furthermore, there have also been links between maternal smoking and adverse neurobehavioral, cardiovascular, respiratory, endocrine and metabolic outcomes in the offspring, which can persist into adulthood [4]. Since rapid organ growth occurs *in utero*, the vulnerability to certain toxins is increased and the exposure to these toxins can potentially interrupt organ development [6]. The inflammatory response and oxidative stress induced by inhaled cigarette smoke may play a key yet common role in all organ disorders by both direct and *in utero* exposure.

Smoking prevalence remains high mainly due to nicotine, which activates the reward pathway, leading to addiction and causing withdrawal symptoms upon cessation from chronic use [7]. New nicotine delivery systems such as electronic cigarettes (e-cigarettes) and heat-not-burn products were initially designed to aid with smoking cessation by delivering nicotine with less toxic chemicals than combusted tobacco leaves [8]. E-cigarettes heat an e-liquid (which may or may not contain nicotine and/or flavorings) to deliver an aerosol (e-vapor) to the user [9]. Heat-not-burn tobacco products heat tobacco leaves at a lower temperature than burning cigarettes but still produce smoke containing nicotine and other chemicals [10]. These devices replicate the oral inhalation and exhalation, taste, rapid systemic delivery of nicotine, hand-to-mouth feel and throat hit sensations (depending on the temperature) that are similar to smoking tobacco cigarettes [8,11,12]. With increasing e-cigarette use, especially as a form of nicotine replacement among pregnant mothers and as recreational usage among the younger generation (including women of childbearing age) [13], there is an urgent need to investigate the intrauterine response to maternal usage of e-cigarettes. This review will summarize the current understanding of the effects of nicotine administration, direct cigarette smoke exposure and e-vapor exposure on fetal development to inspire future research into fetal programming by maternal e-vapor or heat-not-burn exposure.

## 2. Maternal Smoking and Fetal Outcomes

Maternal smoking during pregnancy is an established risk factor for a range of gestational complications and organ disorders in the offspring, including brain and respiratory inflammation, kidney disease, obesity, hyperlipidemia, insulin resistance, and type 2 diabetes [14]. Rapid organ growth occurs *in utero*, increasing the vulnerability of the fetus to certain toxins [6]. Animal studies have shown that the activation of nicotinic acetylcholine receptors (nAChRs) within fetal lungs and brain by nicotine during critical developmental periods impairs normal organ development [15,16,17]. Nicotine inhaled from tobacco smoke during pregnancy can concentrate in the developing fetus (15% more than maternal blood levels), directly impacting fetal intrauterine and postnatal development [18]. However, it is important to recognize that it is not possible to discern the effects of nicotine from the 4000 other chemicals in tobacco smoke, while at the same time the effects of nicotine should not be ignored, especially in e-cigarettes [19]. We will discuss the role of maternal smoking on the offspring’s health status, including evidence of how the fetal outcome change with different forms of nicotine delivery, to inform the reader about new inhaled nicotine delivery systems.

### 2.1. Intrauterine Growth Restriction

Smoking during pregnancy has been linked to low birth weight and the underdevelopment of certain organs, such as the liver and kidneys [5,20]. Of babies with low birth weight, approximately 20% are due to maternal smoking during pregnancy [21,22]. Maternal smoking during pregnancy decreases birth weight and leads to preterm birth in a dose-dependent manner [23,24,25]. Two mechanisms have been traditionally proposed to explain the low birth weight caused by maternal smoking. Firstly, carbon monoxide may lead to hypoxia, decreasing the amount of oxygen reaching fetal tissue. Secondly, nicotine can lead to vasoconstriction of uteroplacental vessels, reducing the delivery of both nutrients and oxygen to the fetus [26]. As a result, nutrition is re-distributed to prioritize vital organs, such as the heart and the brain, at the cost of less vital organs, such as the liver, kidneys, adrenal glands, and pancreas, leading to underdevelopment and functional disorders later in life [20,27,28]. However, at birth, the brain weight of offspring from mouse mothers exposed to cigarette smoke was much smaller than those from sham exposed mothers [29]. There may also be a third mechanism, which is driven by increased oxidative stress. We have shown that maternal smoking can cause oxidative stress in multiple organs, which can persist until adulthood; whereas maternal supplementation of antioxidants during gestation and lactation concominant with tobacco smoke exposure can reverse such impacts on fetal underdevelopment [29,30,31,32]. Interestingly, previous studies also suggest that *in utero* exposure to air pollution alone can lead to low birth weight [33,34,35]. Although not well studied in the literature, certain components in polluted air, such as particulate matter with an aerodynamic diameter ≤2.5 µm (PM_2.5_), have been shown to cause oxidative stress in the placenta whereas heavy metal was shown to directly increase fetal oxidative stress [36]. This raises the question whether nicotine plays such a critical role in fetal underdevelopment induced by maternal tobacco smoke exposure.

In contrast to animal studies where maternal nicotine administration restricted fetal growth, nicotine replacement therapy (NRT) during pregnancy is not linked to low birth weight [37]. The birth weight of newborns from smoking mothers who switched to NRT during pregnancy was similar to those from non-smokers and those who quit smoking during pregnancy [38]. Two studies have reported that NRT usage among pregnant smokers increased birth weight compared to placebo controls, probably due to the effect of nicotine in NRT to suppress appetite being weaker than that due to tobacco smoke inhalation [39,40]. Furthermore, offspring from pregnant mothers using NRT during pregnancy had similar infant survival rates and slightly less developmental impairment (27%) at birth compared to the placebo group (35%) [41]. However, epidemiological evidence on the risk of adult-onset disease in offspring born to mothers using NRT during pregnancy is limited [25,41], and these studies failed to consider the changes in smoking behavior after NRT and the duration of NRT during pregnancy [42].

### 2.2. Lung Health

Epidemiological studies of infants exposed to tobacco smoke during the intrauterine period have found reduced lung function (respiratory compliance, forced expiratory flow, and tidal breathing volume) soon after delivery [43,44,45], which can persist into adulthood [46]. Children born to smoking mothers have an increased risk of developing wheezing, asthma, bronchitis and hospitalizations for respiratory infections [47,48], and the risks are greater in comparison to those arrising from postnatal tobacco smoke exposure [49,50]. The effects of tobacco smoke upon lung health can be partially attributed to low birth weight commonly observed in babies of smoking mothers. In particular, in children that are born premature or small for gestational age, their lungs can be underdeveloped and the normal lung growth does not occur in childhood, resulting in impaired lung function in adults. As such, prematurity or small for gestational age is linked to an increased risk of asthma and reduced lung function in later life [51,52] which acts synergistically with intrauterine smoke exposure to increase the risk of asthma [53].

Using rodent models of maternal smoking (either direct tobacco smoke exposure or nicotine administration), we and others have demonstrated chronic oxidative stress and inflammation in the offspring and detrimental effects on lung development [48,54]. Similar adverse effects have been seen in lung function [55,56]. Direct cigarette smoke exposure in pregnant mothers can activate the receptors for advanced glycation end products (RAGE) and its pathway elements including nuclear factor-κ light chain enhancer of activated B cells (NFκB) and protein kinase (MAPK) family consisting of extracellular signal-regulated kinase-1/2 (ERK1/2) and c-JUN NH2-terminal kinase (JNK) [54]. RAGE are multi-ligand receptors abundantly localised in the lung, responding to by-products of reactive oxygen species (ROS) as produced during oxidative stress and/or pro-inflammatory responses. RAGE signalling is also a key regulator of inflammation in cigarette smoking-related pulmonary diseases. Not surprisingly, the pro-inflammatory cytokines IL-1β and TNFα, as well as oxidative stress response were also increased in the lungs from offspring of mothers exposed to cigarette smoke during pregnancy [54].

In humans, NRT during pregnancy seems to be only related to increased risk of congenital respiratory abnormalities in the offspring when compared to smoking without NRT during pregnancy and non-smoking condition [57]. However, this was only found in one sudy in which the authors also noted that it was underpowered to make these conclusions: prior smoking history was not recorded, and; NRT users were more likely to have asthma—which is inheritable—compared to all other groups [57]. Prenatal nicotine exposure in primates resulted in decreased lung size and volume, increased type I and type III collagens, decreased elastin in the lung parenchyma, increased alveolar volume and increased airway wall area [19]. This impact can even be extended to the second generation offspring, indicating that an epigenetic mechanism may be at play [19]. In addition, exposing pregnant monkeys to nicotine leads to increased expression of α7 nAChRs in the airway epithelial cells and fibroblasts of fetal lungs [58]. Similarly, pre- and postnatal nicotine exposure in a mouse model also decreased forced expiratory flow, which was absent in α7 nAChR knockout mice [16]. Overall, this suggests that the action of nicotine on α7 nAChRs could play a key role in mediating altered lung development due to *in utero* cigarette smoke exposure.

### 2.3. Metabolic Disorders

Cigarette smoke exposure of mothers during gestation and lactation can lead to insulin resistance and glucose intolerance in the adult offspring independent of the postnatal diet [30,59]. In mice offspring from mothers exposed to cigarette smoke during pregnancy, there is a significant increase in inflammation from weaning until adulthood, with alterations in lipid and glucose metabolic markers [60]. Furthermore, *in utero* cigarette smoke exposure is associated with increased circulating triglycerides, which is also a risk factor for systemic insulin resistance by disturbing insulin signalling in the glucose depositing organs, such as skeletal muscle and fat tissue [60,61,62].

Barker’s theory suggests that intrauterine undernutrition permanently alters energy metabolic function in the body [63], classically represented by the Dutch Famine winter [64]. Multiple epidemiological and animal studies have revealed a strong inverse relationship between birth weight and the risk of developing abdominal obesity and metabolic syndrome in adulthood [64,65,66,67]. Thus, fetal undernutrition caused by maternal smoking during pregnancy could also program the offspring to survive in a nutrition scarce environment. However, this incorrect environmental forecast could promote obesity and other related metabolic complications in the offspring later in life [68], due to the adaptations to limit cell numbers in key metabolic organs (e.g., pancreas, liver and skeletal muscle) [69]. These changes in the fetal period will have sequelae that are potentially disadvantageous in the long term by altering hormonal regulation, which increases the prevalence of type 2 diabetes and coronary heart disease [69]. The mechanisms underlying the association between intrauterine growth retardation and the appearance of the metabolic syndrome are still under investigation. Postnatal catch-up growth is common in children with intrauterine growth restriction caused by intrauterine smoke exposure, especially among early pregnancy smokers [70,71,72]. At three years of age, there is a direct link between smoking during early pregnancy and increased body mass index in the offspring without changes in height [73]. This body weight increase continues until 16 years old, with increased fat mass mostly contributing to this phenotype [29]. Interestingly, the first trimester of pregnancy is the most critical period in determining the long-term phenotype in the offspring [74]. However, smoking cessation before pregnancy mitigates such effects [73], reinforcing the importance of the intrauterine environment itself on fetal and early postnatal development.

Animal research using maternal nicotine exposure showed a direct adverse impact on pancreas development by reducing endocrine pancreatic islet size and number [75]. This was accompanied by a decrease in gene expression of specific transcription factors and blood glucose regulating hormones such as insulin and glucagon [75]. As a result, rats exhibited significant pancreatic dysfunction and glucose intolerance [75]. Indeed, several animal studies have reported insulin resistance in adult offspring due to maternal nicotine exposure [15,75,76,77].

### 2.4. Neurological Outcomes in Offspring

Human studies have shown that maternal smoking can also cause long-lasting adverse effects on the structural or functional development of the fetal brain, leading to cognitive disorders [78]. In human studies, offspring from smoking mothers showed significant differences in verbal and visual memory at 13–16 years old [79]. Heavy smoking (>20 cigarettes/day) during pregnancy can also increase the risk of internalising behaviors such as anxiety, fear and sudden mood changes in young children (1.5 and 3 years old) [80]. In addition, maternal smoking increases the risk of attention deficit hyperactivity disorder in a dose-dependent manner [81] and has also been linked to psychological changes such as aggressiveness, decreased social behavior, and depression in the offspring [82,83,84].

Due to the difficulties in accessing human tissues, the understanding of the molecular mechanisms, including inflammation and oxidative stress, in the brain relies heavily on animal modelling. In addition, confounding factors such as paternal smoking, alcohol consumption, socio-economic status, and family history of mental issues can result in inconsistent human findings [80]. Therefore, animal models have the advantage of being able to focus on a single factor in a tighly controlled enviroment. ROS produced by burning tobacco are not removed by the cigarette filters, leading to the activation of inflammatory pathways (e.g., NFκB pathway) in various myeloid and lymphoid cells [85]. ROS can also activate macrophages which further produce more ROS [86]. In adult male offspring of mothers exposed to cigarette smoke, brain inflammation (IL-6, IL-1α receptor and Toll-like receptor (TLR)4 expression) is increased [29]. The activation of TLRs stimulates the production of IL-1β and IL-6 in monocytes, which in turn enhances TLRs expression via a positive feedback loop [29]. Neuroinflammation plays a crucial role in the development of neurodegeneration. Increased levels of TLR4 and IL-1 can both lead to an elevation of β-amyloid, leading to the development of Alzheimer’s disease. Increased brain IL-6 levels are also associated with increased anxiety, autism-like behavior, and the progression of neurodegenerative diseases [87]. Indeed, increased severity of schizophrenia or autism has been found in offspring of smoking mothers who have high levels of circulating inflammatory cytokines [88,89].

Although the brain receives priority nutrition delivery, smoking during pregnancy is closely linked to small brain weight, and frontal lobe and cerebellar volumes [90]. This has been suggested to be due to vasoconstrictor properties of nicotine, which can reduce blood flow to the placenta [90]. In addition, cigarette smoking can increase carboxyhemoglobin levels during pregnancy which can reduce the oxygen carrying capacity of both fetal and maternal red blood cells [91]. The outcome is hypoxic-ischemia (HI) which diminishes the exchange of oxygen and carbon dioxide and causes severe lactic acidosis [78]. HI itself is one of the causes of cerebral palsy and associated disabilities in children [92]. Earlier studies showed that maternal smoking causes hypoxia in the fetus of rhesus monkeys [93]. Smoking ten or more cigarettes per day during pregnancy has been shown to increase the risk of cerebral palsy which is a well-known outcome of HI encephalopathy [94].

In animal models, nicotine has also been shown to activate nicotine acetylcholine receptors (nAChR) in the brain, which are important in regulating brain development. Nicotine exposure during the first trimester of pregnancy (2 mg/kg/d) leads to structural changes in the hippocampus and somatosensory cortex in rats [95]. However, human consumption varies between 3.7 to 67.1 mg/day [96], suggesting unphysiological exposure in animal models is needed to achieve the desired effects. As such, when nicotine dose was reduced to 1.5 mg/kg/d, it no longer seemed to change the brain size in the offspring [97]; whereas maternal smoking reduces the brain size at birth in humans [29], further supporting the rationale to use NRT or e-cigarette during pregnancy to prevent adverse fetal outcomes. Indeed, NRT usage during pregnancy did not alter the head circumference of the offspring in human studies [39,98]. However, it has been found that offspring from mothers using NRT during pregnancy did not change the risk of attention deficit hyperactivity disorder compared to those who smoked during pregnancy [38], indicating that nicotine could mediate this impact.

### 2.5. Renal Outcomes in Offspring

Maternal smoking, particularly in the first trimester, imposes a significant adverse impact on fetal renal development that increases the risk of developing chronic kidney disease. Although smoking itself is well known to increase the risks of renal dysfunction and chronic renal disorders [99], current understanding on the direct impact of maternal smoking on renal disorders in offspring mostly arises from mouse models of maternal smoking research led by our team [28,29,30,31,32,54,60,100]. Only recently, human studies have identified the link between maternal smoking during pregnancy and the appearance of albuminuria (a hallmark for kidney structure damage) in three-year-old children [101], suggesting impaired kidney function and high risk for chronic kidney disease later in life. This is similar to what we have found in mice [28].

We have comprehensively studied the detrimental impact of prenatal cigarette smoke exposure on renal health during early postnatal developmental period and adulthood, where we identified several mechanisms that play critical roles in the delayed early renal development and adulthood renal dysfunction. These include (1) epigenetic modification of fetal nuclear/mitochondrial DNA; (2) changes in fetal renal growth factors (e.g., Glial-cell-Derived Neurotrophic Factor); and (3) oxidative stress and mitochondrial dysfunction [28,30,32].

Intrauterine growth restriction may hold the key to renal developmental disorders and later onset of renal disorders in offspring of smoking mothers. Fetal and infant kidney volumes and glomerular number significantly correlate with birth weight [28,102,103,104,105]. Newborns with low birth weight have 30% fewer nephron numbers than those with normal birth weight, associated with large glomerular size to restore the function [106] and kidney growth restriction during the first 18 months of life [107]. If glomeruli are overly enlarged, glomerular hypertension and hyperfiltration will occur, causing accelerated nephron loss [108]. This can ultimately lead to glomerulosclerosis, further reducing nephron function [109]. The direct consequences are increased susceptibility to developing hypertension and chronic renal failure [110,111]. Hence it is suggested that maternal smoking may directly lead to impaired renal function, due to a multiplicity of factors, including fewer nephrons, secondary hyperfiltration, and eventually glomerulosclerosis in the long term [28].

In vivo and in vitro evidence suggests that among all chemicals in the cigarette smoke, nicotine could play a role by inducing renal oxidative stress and mtDNA damage in human tissue [112,113,114]. Nicotine is highly concentrated in the kidneys since it is excreted by glomerular filtration and tubular secretion. As such, it has also been considered as a major contributor to kidney disease in smokers [114]. Nicotine alone can cause renal dysfunction in humans and animal models [105,115], attributed to the vasoconstrictive effect of nicotine [105,115], increased activity of the renin-angiotensin-aldosterone system [116], and an increased ratio of the angiotensin type 1 receptor versus type 2 receptor density [105,115]. As nicotine passes the blood-placenta barrier, maternal smoking during pregnancy inevitably affects the kidney health of the unborn child [100]. Nicotine infusion in rat dams also led to smaller kidneys in offspring [117,118], similar to what we have observed in an animal model using direct cigarette smoke exposure [28].

## 3. Difficulties of Smoking Cessation

Due to the negative health impacts of smoking, pharmacotherapies (NRT, bupropion and varenicline) have been developed to aid in smoking cessation by managing nicotine addiction and withdrawal symptoms [119]. NRT provides an alternate nicotine source, enhancing pleasure and reducing withdrawal symptoms [120]. Bupropion acts by inhibiting dopamine uptake in the brain, thereby reducing the pleasurable dopaminergic effects of nicotine. Furthermore, it acts as an antagonist on a wide range of nAChRs, blocking positive responses to nicotine administration [121]. Varenicline is an α4β2 nAChR partial agonist, with a higher affinity than nicotine [119]. However, only about 25% of smokers who use these medications are able to stop smoking for over six months [122].

Unfortunately, smoking cessation therapies may not always be useful during pregnancy [123]. A Cochrane Review found that NRT was not different from placebo to promote smoking cessation during pregnancy [119]. This may be due to increased nicotine metabolism during pregnancy or the low adherence rates of the intervention. Additionally, there is insufficient evidence on the safety and efficacy for the use of bupropion and varenicline during pregnancy [119]. Even among pregnant women who do manage to quit, a high proportion tend to relapse [123], with half of the women who quit during pregnancy relapsing within four months of delivery [124]. Pregnant women who wish to quit smoking but are unable, are left with few options and may be drawn to heat-not-burn devices or e-cigarettes [48].

## 4. Heat-Not-Burn Tobacco Smoking

The term “heat-not-burn” refers to tobacco heated (at ~350 °C) by an electrically-powered element or carbon, not combusted (at ~800 °C) [10]. Heat-not-burn devices are currently sold in Japan and Europe, but are globally not as popular as the e-cigarette. Philip Morris International Inc. (New York, NY, USA), developed the iQOS, currently the most popular device, and claims it produces no second-hand smoke [125]. Research suggests that this type of device delivers similar or less nicotine to the user compared with filter top tobacco cigarettes, whereas the toxin levels such as tobacco-specific nitrosamines were reduced to one fifth and carbon monoxide was one hundredth, as well as other carcinogenic chemicals [10,125,126,127,128,129]. However, it generally induced less satisfaction compared with the conventional tobacco cigarette [127,130,131,132].

The efficacy of nicotine delivery has been studied in the smoke generated by the device and in human users [125,133,134]. However, the physiological changes, such as inflammation in multiple organ systems, energy metabolism, and carcinogenesis, in responses to heat-not-burn tobacco smoke has not been well characterized due to limited research in this area, especially in animal models. An *in vitro* study suggested a less harmful pathophysiological response in human organotypic oral epithelial cultures when exposed to such smoke [135]. An animal study showed that heat-not-burn tobacco smoke did not increase surfactant lipids, surfactant proteins, surfactant metabolizing proteins, inflammatory eicosanoids and their metabolic enzymes, and several ceramide classes, compared with tobacco cigarette smoke-exposed mice [136]. Even with reduced toxins in heat-not-burn tobacco smoke, overuse (40 cigarettes/day) can still lead to eosinophilic pneumonia in humans [137]. There is insufficient evidence on the efficacy of heat-not-burn tobacco on smoking cessation. Neither is there information on the potential impact of maternal inhalation of heat-not-burn tobacco smoke during pregnancy on fetal outcomes, all of which require urgent attention.

## 5. E-Cigarette Vaping

### 5.1. Rise in Popularity among the Young and Pregnant

Among developed nations, tobacco cigarette smoking has recently plateaued after declining since the 1960s [4]. Meanwhile, e-cigarettes have rapidly infiltrated the market since their development in 2004 [8], with an estimated 3.5 million devices sold in 2012 [138]. E-cigarettes’ popularity among smokers arises from the heavily advertised notion that they are safer and cheaper than tobacco cigarettes, despite the lack of solid scientific evidence on their pathophysiological impacts [13]. Although e-cigarettes were designed as a smoking cessation aid, they are largely used recreationally. Product design and advertising often target youth [8,19], leading to increased usage among US high school students which doubled in just one year, from 2011 (4.7%) to 2012 (10.0%) [139]. After reaching a peak in 2015 (16.0%), usage plateaued in 2016 (11.3%) and 2017 (11.7%) [139,140,141,142].

E-vaping among pregnant women is increasing since they are often perceived to be a safe and effective form of smoking cessation [13,143,144]. Furthermore, e-cigarette usage among women of reproductive age is increasing [26], which is especially concerning considering a large number of unplanned pregnancies [145]. A recent online survey found that pregnant women were more likely to be e-vapers (6.52%) or dual users (8.54%) compared to cigarette smokers (5.62%) [146]. An additional survey of pregnant women found that 28% had used e-cigarettes during pregnancy, while 7% were current e-cigarette users and 3.4% were daily e-cigarette users [147].

However, this rise in popularity has been met with controversy, stemming from a lack of scientific safety evidence [148]. On one hand, e-cigarettes could be a revolutionary replacement for tobacco cigarettes, reducing the current health and economic burden associated with tobacco smoking [19,149]. On the other hand, they may reinvigorate and renormalize nicotine addiction, effectively undermining worldwide efforts to reduce the rates of tobacco smoking [13,150].

### 5.2. E-Cigarettes for Smoking Cessation and Replacement

E-cigarettes are often used for smoking replacement or cessation aid due to perceptions of safety and efficacy [8]. However, adequate efficacy evidence is limited, especially when compared to existing therapeutic options. A 2016 Cochrane review concluded that nicotine-containing e-cigarettes were more effective compared to nicotine-free e-cigarettes, but similar to nicotine patches for long-term smoking cessation [12]. Unfortunately, the overall quality of the evidence was rated low since there were only a small number of low-powered studies with large confidence intervals [12]. The comparison between nicotine-containing and nicotine free e-cigarettes neglected existing therapeutic options [151]. A randomized control trial of 657 participants found that e-cigarettes were similar in their effectiveness in assisting with smoking cessation compared to nicotine patches [152]. However, this study may overestimate the efficacy of e-cigarettes since they were delivered directly to the participants, whereas those in the nicotine patch group were given a voucher which needed to be redeemed at a pharmacy [12]. If e-cigarettes are to be used as a therapeutic device, their efficacy must be established against existing options such as NRT, varenicline and buproprion [48]. Furthermore, e-cigarettes produce more toxins than other forms of NRT and are likely to be more harmful [153].

### 5.3. Toxins in the E-Vapor

E-cigarettes use a battery-operated atomizer to heat e-liquid within a tank to produce an aerosol (e-vapor), delivering nicotine and flavorings to the user [9]. E-liquids often contain propylene glycol and/or vegetable glycerin, nicotine (0 mg/mL to 24mg/mL (or more)) and flavorings. Flavoring compounds have been generally regarded as safe as food additives by the Food and Drug Administration, leading to perceptions of safety; however they may not be safe for inhalation [8]. In fact, a Polish study found that 15% of adult e-cigarettes users believed e-cigarettes were ‘absolutely safe’ [154]. Public Health England’s claim that e-cigarettes are 95% safer than tobacco cigarettes has been widely distrusted by the media [155]. However, this analysis has been criticized for being an unsubstantiated estimation based on the opinions of a group of individuals without any tobacco control expertise [156]. The long-term biological effects of e-cigarette are still unknown, especially among vulnerable populations [8] considering the long latency of many chronic diseases such as type 2 diabetes and chronic obstructive pulmonary disease [150]. In addition, out of the approximately 7000 different flavored e-liquids available [157], only a handful have been adequately examined.

Differences in e-cigarette heating capacity and puffing behavior alter the vaping experience and in particular the delivery of nicotine [13,158]. The delivery of nicotine at high temperatures produces what is called a throat hit, which replicates the sensation of cigarette smoking. However, heating e-liquids to high temperatures produce more toxins, such as formaldehyde hemi-acetyls [159]. Propylene glycol and vegetable glycerin are often used as humectants, aerosolizing the nicotine and flavorings when heated. These humectants can be oxidized to produce toxic carbonyl compounds, formaldehyde, acetaldehyde and acrolein [160,161], which have been detected in the e-vapor as well as in the exhaled breath after e-vaping [162]. Formaldehyde is classified as a human carcinogen; acetaldehyde is a possible human carcinogen, and; acrolein may contribute to cardiovascular disease [153]. The carcinogenic tobacco-specific nitrosamines have also been detected in the e-vapor [153]. The levels of these contaminants are often associated with nicotine levels within the e-liquid. Other volatile organic compounds and heavy metals have been detected in e-vapor which may also pose health risks [163].

Even though many flavoring compounds have been demonstrated to be safe for oral ingestion, they are converted to toxic and carcinogenic by-products via incomplete combustion during e-vaping [164]. Many of the flavoring compounds are aromatic aldehydes and produce toxic chemicals at high temperature [165]. Benzaldehyde, a respiratory irritant [166], was detected in the e-vapor generated from 108 out of 145 flavored nicotine e-liquids, with the highest levels detected in cherry flavorings [167]. In addition, diacetyl has been detected in the e-vapor from buttery or creamy flavors [168,169]. Occupational exposure to diacetyl causes bronchiolitis obliterans or “popcorn lungs” [170]; however, there is conjecture as to whether concentrations in the e-vapor are high enough to be of concern [171].

Furthermore, heavy metals such as chromium, cadmium, lead, nickel, tin and copper have been detected in the e-vapor [153,172,173,174,175]. These metals most likely originate from e-cigarette coils which are often either kanthal (iron, chromium and aluminium alloy) or NiChrome (nickel and chromium alloy) [176]. The concentrations of some of these metals were detected at higher levels compared to tobacco cigarette smoke [177] and exceeded current exposure limits [176]. E-cigarettes are reported to be safer than tobacco cigarettes due to the inhalation of fewer and lower doses of toxic compounds, but are by no means completely safe [149,178]. Furthermore, e-cigarettes are more harmful compared to existing NRT. Goneiwitz et al. (2014), compared the levels of certain toxic compounds generated by an e-cigarette to a Nicorette^®^ inhaler. While the e-vapor contained tobacco-specific nitrosamines and volatile organic compounds (toluene and p,m-xylene), the vapor from the Nicorette^®^ inhaler did not [153].

In human studies of acute e-vapor exposure, limited respiratory complications are observed especially when compared to tobacco cigarette smoking [179]. Furthermore, improvements in lung and cardiovascular function have been reported when tobacco smokers switched to e-cigarettes due to less toxin production by the latter than tobacco smoke [180,181,182]. However, short-term e-vaping has also been shown to impair pulmonary function [183] and cardiovascular outcomes such as heart rate and blood pressure, at similar levels to tobacco cigarettes in some cases [184,185,186]. Furthermore, e-vapers had increased pulmonary innate inflammatory responses with changes in markers of neutrophil activity which were overlapping yet distinct from cigarettes smokers [187]. Acute e-cigarette usage among smoking naïve individuals also resulted in changes in the transcriptome of small airway epithelium and alveolar macrophages [188]. In addition, respiratory adverse events such as cough, asthma exacerbations, bronchitis, difficulty in breathing and pneumonia have been reported from passive exposure among non-users [189]. The long-term biological impacts of e-vaping are currently unknown, especially among vulnerable populations.

### 5.4. Inflammatory and Oxidative Stress Response to E-Vapor Exposure

Exposure of animals to e-vapor has been shown to increase respiratory inflammation and oxidative stress. E-vapor also contains ROS however at a lower level than cigarette smoke, with 1.2–8.9 nmol H_2_O_2eq._/puff. This could be derived from the pyrolysis of the nicotine, humectants and flavoring agents, along with the catalytic activity of metals [190]. As a result, acute e-cigarette vaping in a crossover, single-blind study in humans increased blood biomarkers of oxidative stress [191]. Additionally, C57BL/6 mice acutely exposed to e-vapor (with nicotine, tobacco flavor) showed increased levels of the inflammatory markers IL-6, IL-1α and IL-13 in bronchoalveolar lavage fluid (BALF) and decreased levels of the antioxidant glutathione [192]. Furthermore, another study examined the short-term administration of e-vapor (with nicotine, no flavor) to C57BL/6 mice and found increased granulocytes within the BALF [193]. Increased systemic oxidative stress was also observed reflected by increased levels of 8-hydroxy-2′-deoxyguanosine and nitrotyrosine levels [193]. We have also observed a similar increase in pro-inflammatory cytokines (IL-1β, IL-6, and TNFα) and RAGE pathway elements in mice with long-term exposure to e-vapor (with and without nicotine, tobacco flavor), with some of the changes even being nicotine-independent, supporting the toxicity of the solvent and flavorings [194]. Direct e-vapor exposure alters respiratory inflammatory and ROS response in mice [195]. As e-cigarettes are relatively new in the market, there is still a lack of evidence on their long-term impact.

Evidence also suggests that flavorings within the e-liquid can be cytotoxic even without vaporization. Behar et al. (2014) have demonstrated that e-liquids (cinnamon flavor, with and without nicotine) are cytotoxic to human embryonic stem cells and human pulmonary fibroblasts cells [196], with cinnamaldehyde and 2-methoxycinnamaldehyde as being the most cytotoxic components [196]. Human embryonic stem cells were more sensitive to the damage by the e-liquids than human pulmonary fibroblasts cells, suggesting potential fetal toxicity if inhaled during pregnancy [196].

### 5.5. Maternal Vaping during Pregnancy and Potential Harmful Effect on the Offspring

E-cigarette usage is increasing rapidly in pregnant women and in women of reproductive age, increasing the risk of *in utero* e-vapor exposure [146].

Nicotine is the main psychoactive component in cigarette smoke, being highly addictive and the reason cigarette cessation is difficult [120]. In human studies, nicotine is not used in isolation, so many studies are confounded by previous or current cigarette smoking status. Multiple animal studies have demonstrated that nicotine is a developmental toxin, and is responsible for many of the health consequences associated with *in utero* tobacco smoke exposure [48]. However, this may not represent the whole story of the potential impact of maternal e-vaping on the health outcome in the offspring due to the high doses used in the animal studies. In humans, nicotine alone does not necessarily affect fetal development, as several studies have shown that NRT during pregnancy is not linked to low birth weight [37,38]. Therefore, it is the combination of nicotine and other chemicals in tobacco cigarette smoke or e-vapor that can cause an adverse impact on fetal development. The other toxic components from heated e-vapor have not been adequately studied during pregnancy and may also cross the placenta and impact on the fetus, since they have been shown to cause cytotoxicity, inflammation and oxidative stress [192,193,196,197]. Inflammatory response and oxidative stress serve a common mechanism through which maternal smoking causes organ disorders in both young and adult offspring, predisposing them to various disorders. Limited in vitro and in vivo evidence suggests that e-vaping may induce similar impacts in mothers using e-cigarettes and in their offspring through similar mechanisms, as e-vapor components have been shown to cause cytotoxicity, inflammation and oxidative stress [192,193,196,197].

To date, no human study on the impacts of maternal e-vaping during pregnancy on offspring’s health outcomes has been conducted [17]. Animal models indicate that e-vapor exposure during critical developmental periods, especially *in utero* can impair organ development and lead to organ damage. In zebrafish (*Danio rerio*), exposure to e-vapor extract (with nicotine, tobacco flavor) impaired cardiac development [198]. Murine neonatal exposure to e-vapor (with nicotine, no flavor) during the first 10 days of life resulted in larger alveolar size, indicating the inhibition of alveolar growth and potential respiratory disorders [6]. Furthermore, exposure to e-vapor (with nicotine, no flavor) during late gestation and in the early postnatal period increased risk-taking behavior at adulthood [199]. However, not all e-liquids contain nicotine, and many contain flavorings to increase palatability. Exposure of C57/B6 mice to e-vapor (with and without nicotine, tobacco flavor) in early life induced gene changes corresponding to adverse neurobiological and neurobehavioral outcomes [200].

Our own research using direct e-vapor exposure in pregnant mice showed that nicotine-containing e-vapor (tobacco flavor) reduced birth weight compared to those from sham exposed and nicotine-free e-vapor (tobacco flavor) exposed mothers [195]. As discussed in Section 2, a low birth weight correlates with many health complications later in life. Furthermore, we found that maternal e-vapor exposure during pregnancy altered lung pro-inflammatory cytokine levels and DNA methylation, which were independent of nicotine [195]. Therefore, other components in the e-vapor also had significant impacts. In addition, maternal e-vapor exposure worsened the features of allergic asthma in offspring mice with nicotine-containing e-vapor exerting greater effects [201]. Moreover, neurocognitive function was impaired in adulthood offspring, with nicotine-containing e-vapor having a major impact on short-term memory [194]. Changes in brain metabolic regulators were also observed, with alterations in brain inflammation and oxidative stress in the offspring [202].

The impact of maternal e-vapor exposure on other organs has not been well reported in the literature. Further research into the area of fetal programming due to maternal e-vapor is required.

## 6. Conclusions

The impacts of maternal tobacco cigarette smoking on respiratory, metabolic and neurological complications in the offspring have been well documented. Nicotine, along with the oxidative and inflammatory properties of cigarette smoke, seems to be responsible. Recently, e-cigarettes have become increasingly popular, but their prenatal impacts have not been adequately examined. Since they are able to induce inflammation and oxidative stress independent of nicotine concentration, it is likely that they will have advese effects. Murine models have established that maternal e-vapor exposure during pregnancy can affect respiratory and neurological functions. Hence, e-cigarette usage during pregnancy should not be encouraged. Additional studies in human are necessary to support the use of e-cigarette replacement during pregnancy.
